# Histologic findings associated with laser interstitial thermotherapy for glioblastoma multiforme

**DOI:** 10.1186/s13000-019-0794-4

**Published:** 2019-02-15

**Authors:** J. Bradley Elder, Kristin Huntoon, Jose Otero, Behiye Kaya, Jeff Hatef, Mostafa Eltobgy, Russell R. Lonser

**Affiliations:** 1 Department of Neurological Surgery, The Ohio State University Wexner Medical Center, 410 West 10th Avenue, Doan 1047, Columbus, OH 43210 USA; 2 Division of Neuropathology, Department of Pathology, The Ohio State University Wexner Medical Center, Columbus, OH USA

**Keywords:** Glioma, Glioblastoma multiforme, Histology, Laser interstitial thermotherapy, Treatment

## Abstract

**Background:**

Laser-interstitial thermal therapy (LITT) has been supported by some authors as an ablative treatment of glioblastoma multiforme (GBM). Although the effects of LITT have been modeled in vivo, the histologic effects in a clinical circumstance have not been described. We analyzed tissue from a patient who underwent LITT as primary treatment for GBM.

**Case presentation:**

A 62-year-old male was diagnosed with a left temporal GBM and underwent LITT at an outside institution. Despite corticosteroid therapy, the patient was referred with increasing headache and acalculia associated with progressive peritumoral edema two weeks after LITT procedure. *En bloc* resection of the enhancing lesion and adjacent temporal lobe was performed with steroid-independent symptom resolution (follow-up, > 2 years). Histologic analysis revealed three distinct histologic zones concentrically radiating from the center of the treatment site. An acellular central region of necrosis (Zone 1) was surrounded by a rim of granulation tissue with macrophages (CD68) (Zone 2; mean thickness, 1.3 ± 0.3 mm [±S.D.]). Viable tumor cells (identified by Ki-67, p53 and Olig2 immunohistochemistry) were found (Zone 3) immediately adjacent to granulation tissue. The histologic volume of thermal tissue ablation/granulation was consistent with preoperative (pre-resection) magnetic resonance (MR)-imaging.

**Conclusion:**

These findings are the first in vivo in humans to reveal that LITT causes a defined pattern of tissue necrosis, concentric destruction of tumor and tissue with viable tumor cells just beyond the zones of central necrosis and granulation. Furthermore, MR-imaging appears to be an accurate surrogate of tissue/tumor ablation in the early period (2 weeks) post-LITT treatment. Surgery is an effective strategy for patients with post-LITT swelling which does not respond to steroids.

## Background

Laser-induced thermal therapy (LITT) has been used to ablate primary tumors, metastatic lesions and radiation necrosis [1–7], as well as selectively destroy epileptic foci in cases of medically-intractable epilepsy [8–11]. LITT relies on thermal tissue destruction that can be monitored by real-time magnetic resonance (MR)-imaging thermography. While the histologic effects of LITT have been described in vitro, in *naïve* and tumor animal models or inferred from MR-imaging in the clinical circumstance [3, 12–14], direct understanding of its histologic impact in human pathologic states has not been defined in humans. This information is necessary in order to fully evaluate the risk/benefits of LITT compared to classical en bloc resection of surgically accessible tumors. Insight into the effects of LITT on complex anatomic structures, its impact on neoplastic processes and clinical-imaging correlates is critical to understanding this modality.

To define the histologic and biologic effects of LITT, we systematically investigated the impact of this therapy after its use as the primary treatment of a glioblastoma multiforme (GBM). Specifically, we analyzed the histologic findings, effect on tumor viability surrounding treatment region and clinical-imaging correlates in a patient that underwent LITT as the initial treatment for GBM followed by resection 2 weeks later.

## Case presentation

### Initial management

**Fig. 1:**
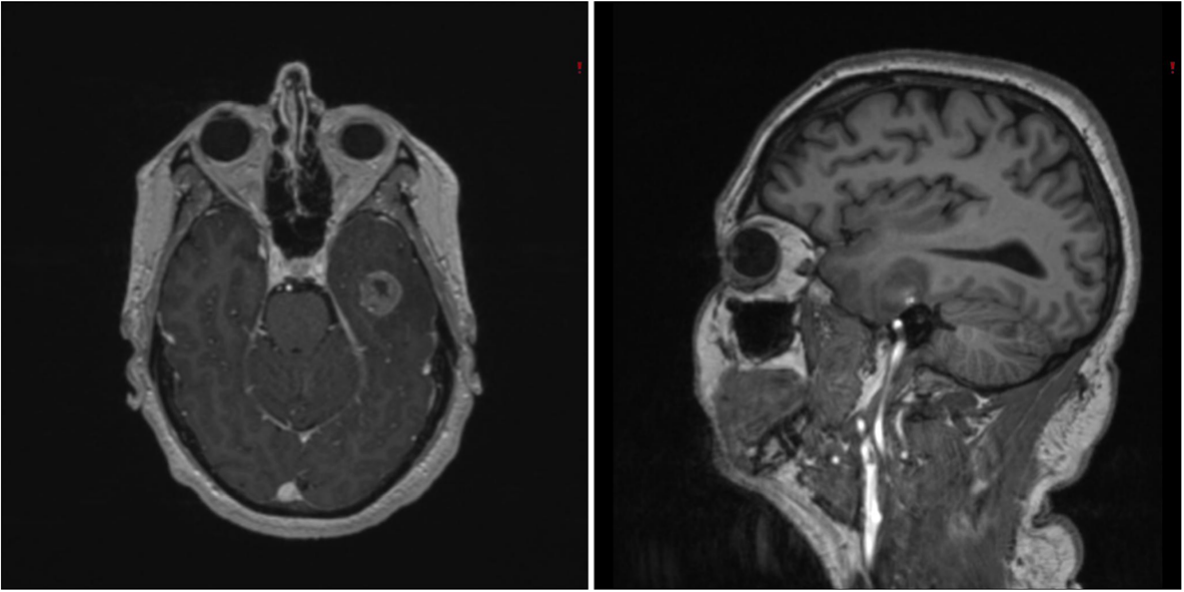
Magnetic resonance (MR)-imaging at initial evaluation at outside institution. T1-weighted post-contrast MR-imaging in the axial (*Left*) and sagittal (*Right*) planes demonstrating a contrast-enhancing lesion (maximum diameter 2.1 cm) that stereotactic needle biopsy confirmed to be glioblastoma multiforme. After biopsy, the patient underwent laser interstitial thermal therapy of the enhancing tumor

This 62-year-old male presented to an outside institution after an acute episode of aphasia. MR-imaging was performed and revealed an enhancing left temporal mass (maximum enhancing diameter, 2.1 cm, Fig. 1). Biopsy of the lesion was histologically consistent with GBM (IDH1R132H negative, 1p/19q intact, MGMT unmethylated, p53 positive and without EGFR amplification). The patient underwent LITT (Energy: 24.42kJ, Pulses: 876, Time: 0:29:07) of the enhancing portion of the GBM via a single treatment track approximately 4 cm in length. The patient was discharged home on post-treatment day 2 at his neurological baseline.

### Open resection

**Fig. 2:**
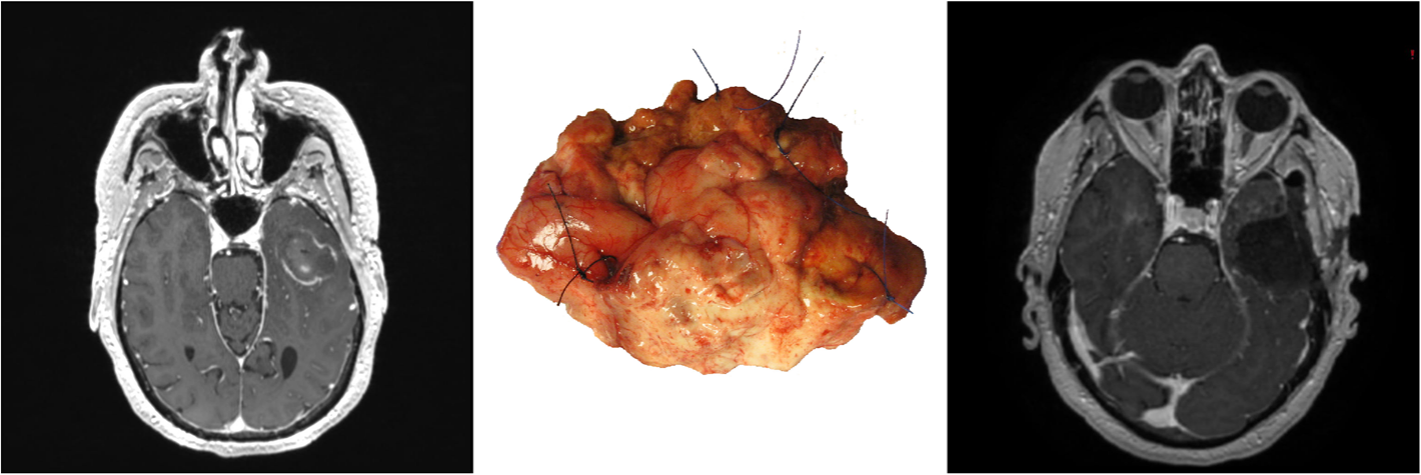
Magnetic resonance (MR)-imaging and pathologic findings at resection of left temporal laser-induced thermal therapy treated glioblastoma multiforme (GBM). Preoperative (before resection) T1-weighted post-contrast MR-imaging in the axial *(Left*) plane demonstrates enhancing necrotic region of treatment/tumor. (*Middle*) *En bloc* resection of the enhancing GBM/treatment site and surrounding tumor-infiltrated tissue (6.5 cm anteroposterior and 4.4 cm mediolateral dimension) was performed. Postoperative T1-weighted post-contrast MR-imaging in the axial (*Right*) plane demonstrates complete resection of the enhancing necrotic region of treatment/tumor

Two weeks after LITT, despite corticosteroid therapy, the patient presented to our institution with complaints of increasing headache and calculation difficulties. MR-imaging revealed tissue necrosis with hemorrhagic material within the LITT treated region and edema surrounding the treatment site. The patient underwent *en bloc* resection of the enhancing lesion and adjacent tumor-infiltrated brain via a left temporal craniotomy (Fig. 2) . Patient headache and calculation difficulties resolved after surgery (discharged post-operative day 2). Post-operative MR-imaging confirmed complete resection of enhancing lesion.

### Clinical outcome

He underwent adjuvant radiation therapy and was treated to a dose of 5945 cGy in 29 fractions with concurrent temozolomide. Subsequent maintenance temozolomide was discontinued after 5 cycles due to persistent pancytopenia.

Interestingly, despite no surgical or medical treatment for the past 4 years, serial MR imaging since surgery has shown no recurrence of his disease.

### Pathologic findings

**Fig. 3:**
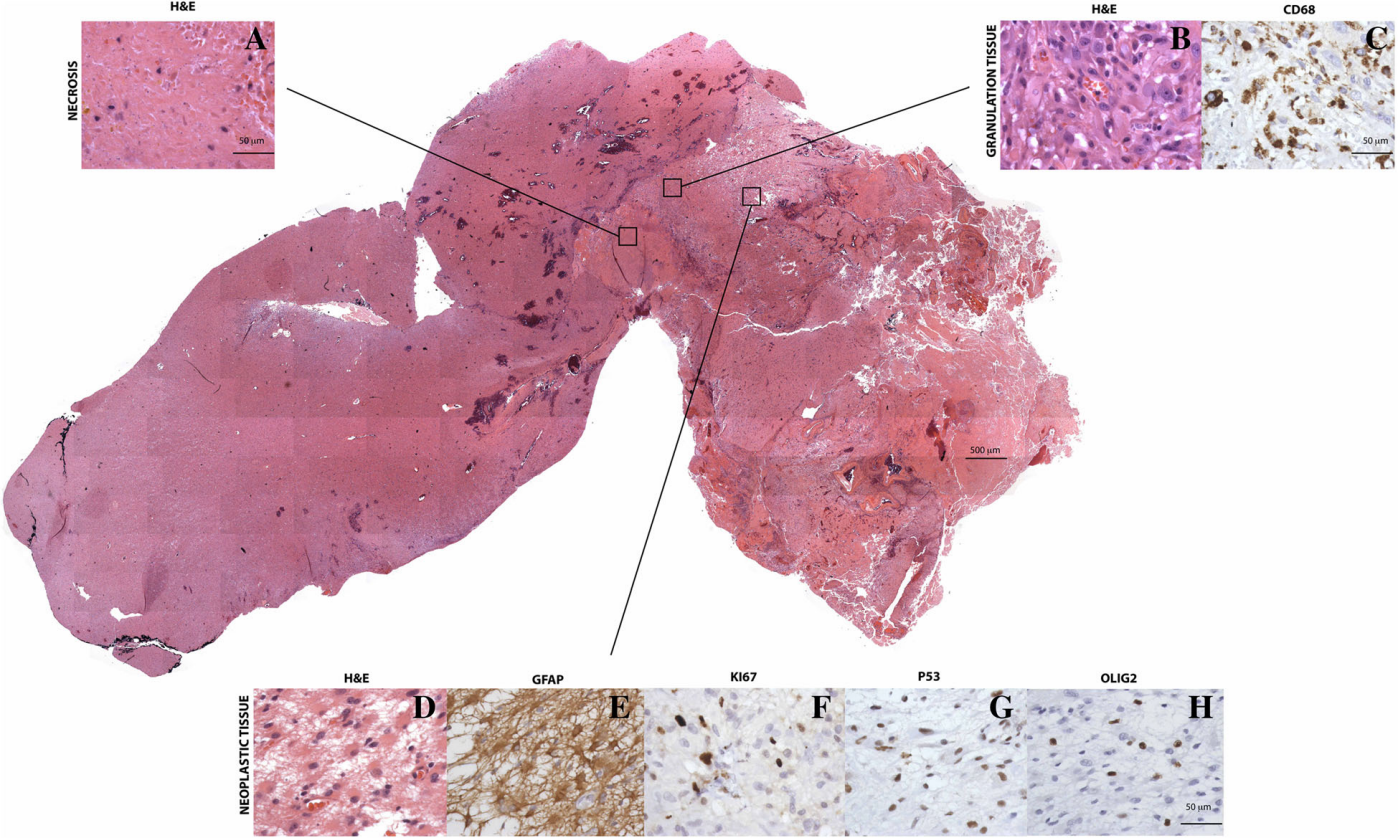
Histologic analysis of laser-induced thermal therapy treated left temporal glioblastoma multiforme revealed distinct histologic zones. Hematoxylin and eosin stained sections represent neoplastic tissue surrounded by granulation tissue and necrosis. Section images were captured (original magnification 4X) and collaged. Higher-magnification of **a** necrotic area (Zone 1), **b** granulation tissue (Zone 2) and **d** tumor (Zone 3). **c** Macrophages detected by CD68 immunohistochemistry in granulation tissue. **e** Glial fibrillary acidic protein stained astrocytes (**f**, **g** and **h**). Single cells with positivity for **f** Ki67, **g** p53 and **h** Olig2 consistent with viable glioma

The gross specimen was 6.5 cm in the anteroposterior and 4.4 cm in the mediolateral dimensions. Histological examination revealed a thermal injury pattern characterized by 3 distinct staining patterns in relation to the different zones of the lesion. . First, a central necrotic zone (Zone 1) devoid of cells was present (Fig. 3a) in which there was gradual loss of staining and early resorptive changes at the margins. Surrounding the necrotic zone, an active rim (Zone 2) of granulation tissue was present (mean thickness, 1.3 ± 0.3 mm) which included vascular proliferation, lymphocytes and microglia positive for CD68 and CD45, respectively, just beyond the necrotic core and mesenchymal and glial reaction at the margin (Fig. 3b-c). Immediately beyond the granulation tissue zone, cytologically atypical, GFAP-positive astrocytes were found (Zone 3) (Fig. 3e). Immunoreactivity in these cells to OLIG2, p53, and Ki67 confirmed their neoplastic nature (Fig. 3f-h). The majority of these tumor cells showed moderately intense immunoreactivity to p53. IDH1^R132H^ was negative in tumor cells by immunohistochemistry.

**Fig. 4:**
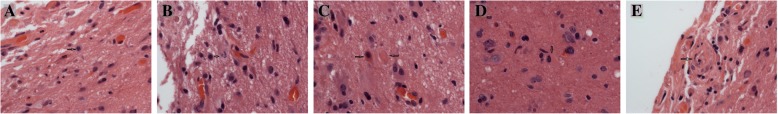
Histologic analysis of laser-induced thermal therapy treated left temporal glioblastoma multiforme revealed showed some hallmark characteristics including a mitotic body (**a**) a multinucleated cell (**b**) axonal spheroid as well as an ischemic neuron (**c**) microglia and reactive gliosis (**d**) and an occluded blood vessel as well as thrombotic vascular changes (**e**)

Additional immunohistochemical examination of the tissue showed hallmark features previously described in non-human tissue in vivo that has undergone LITT. In the area of viable tumor, there were noted mitoses (Fig. 4a, Zone 3). Within the transition area of from the necrotic area to the viable tumor zone was a band of granulation tissue, in which a histiocytic reaction with multinucleated giant cells was present. (Fig. 4b, Zone 2). Axonal ischemic changes such as axonal spheroids were noted (Fig. 4c, Zones 1 & 2). Moreover, cells with nuclear fragmentation indicating neuronal and cell body injury in response to laser ablation were detected (Fig. 4c, Zones 1 & 2). Microglial cells were noted in the areas of high degrees of reactive astroglosis (Fig. 4d, Zone 2). Vascular effects of laser treatment including thrombotic occlusion of vessels were also found. These thrombotic changes were most prominent near the area of laser ablation (Fig. 4e, Zone 1).

Additionally, genomic analysis revealed the following modifications: EGFR L861Q (kinase domain) and no EGFR amplification, R108K (extracellular domain) activating mutations, PTEN G36R (missense mutation within the PTEN phosphatase domain), CDKN2A/B loss**,** IDH1 negative, 1p 19q intact with a Ki-67of 25–30%.

## Discussion

### Previous findings histologic

Prior studies examining features associated with LITT have been performed in animal models [13, 15, 16]. Specifically, defined histologic analyses in naïve animal brains have revealed that LITT creates a central region of tissue necrosis with tissue breakdown products surrounded by gliosis and edema. Histologic analysis of LITT effects in pathologic states, performed exclusively in rodent models of glioma and metastasis, has revealed that LITT ablates tumor cells/tissue resulting in similar features of central necrosis with tissue breakdown, granulation tissue formation and surrounding edema. While previous work has shown that tumor (ependymoma) control can be correlated with thermal damage on MR-imaging [7], the histologic mechanisms of tumor ablation in a patient have not been defined in humans.

To define the histologic features associated with LITT for tumor (GBM), we describe the tissue effects of this therapy in a clinical case of GBM that was not treated with other adjuvant modalities. These findings provide critical insights into the biologic, clinical and histologic features of this therapeutic modality.

### Current case

#### General features

The circumstances in the current case provided a unique opportunity to better understand the histologic features of LITT for GBM. Specifically, the use of LITT as the primary treatment for GBM in this patient provided the prospect to examine the histologic features of this therapy without the confounding effects adjuvant therapy (chemotherapy, radiation or immunotherapy). The fact that the tumor was in a surgically accessible region that permitted the *en bloc* resection of the enhancing lesion and surrounding tumor-invaded brain tissue permitted comprehensive assessment of the pathologic/therapeutic effects of LITT in GBM. Our analysis of the histopathologic changes of the LITT area of the tumor and surrounding tumor are similar to what has been reported in mice modeling except that the degree of reactive changes surpassed what had been seen in the past. For these reasons, insights from this case can critically inform biologic and therapeutic understanding of LITT for GBM.

#### Clinical findings

The clinical findings in the current case reveal important characteristics associated with LITT for GBM. Previously, LITT has been associated with well-described progressive post-treatment tumor-tissue edema on MR-imaging. Imaging data indicate that the post-LITT edema develops within 3 days of treatment and can persist for several weeks (up to 6 weeks) [12, 17, 18]. Similar to other tumor-associated pathologic states [19], imaging data indicate that the post-LITT edema may be the result of treatment-related increased vascular permeability [18]. *En bloc* resection of the tumor as well as a region of associated edema and gliosis performed resulting in elimination of the source of edema and steroid independence.

#### Treatment implications

Features of the current case provide several treatment insights. First, surgical resection can be used to effectively manage post-LITT swelling refractory to corticosteroid therapy. Removal of the LITT treated volume and surrounding tissue/tumor region results in immediate improvement in tissue swelling and permits corticosteroid independence. Second, the findings support the use of MR-imaging in the early treatment period (2 weeks) to accurately define the volume of effective tumor treatment. *En bloc* resection of the tumor and surrounding region allowed for the precise anatomic and volumetric quantification for comparison to MR-imaging in this case. Finally, critical analysis of the effectiveness of LITT (compared to resection) and defining optimal regions for GBM treatment will be essential moving forward. Finally, this patient has survived 55 months as of this report without recurrence. If additional evidence emerges supporting a synergistic effect of LITT prior to surgical resection, then additional clinical studies may be warranted.

#### Histologic findings

Similar to animal histologic studies, the current case reveals that LITT creates concentric non-specific tissue destruction extending approximately 1.5 cm (radius) perpendicular from the thermal source. Consistent with the tissue ablation profile, histologic analysis showed that tumor cell destruction was most effective nearest to the LITT source. Likewise, viable GBM cells were found immediately adjacent to the granulation zone surrounding the necrotic core induced by the thermal therapy. Histologic findings correlated with MR-imaging features, including areas of thermal-induced tissue destruction that appeared as a rim of contrast-enhancement surrounding a core of T2-weighted hyperintensity.

#### Limitations

The main limitation of this manuscript is the inherent limitation of being a single case reporting pathological findings that may or may not be similar to other cases of early acute post-LITT findings.

## Conclusion

LITT causes a defined pattern of tissue necrosis characterized by concentric destruction of tumor and tissue, with viable tumor cells just beyond the zones of central necrosis and granulation. MR-imaging is an accurate surrogate of tissue/tumor ablation in the early period (2 weeks) after treatment. Swelling caused by LITT can be effectively treated with surgical resection of the lesion. Further histologic studies are needed to more fully characterize the immediate, intermediate and long-term effects of LITT on various intracranial pathologies.

## Abbreviations

GBM: Glioblastoma multiforme
LITT: Laser-interstitial thermal therapy
